# Effects of *Lactobacillus plantarum* on the Fermentation Profile and Microbiological Composition of Wheat Fermented Silage Under the Freezing and Thawing Low Temperatures

**DOI:** 10.3389/fmicb.2021.671287

**Published:** 2021-06-09

**Authors:** Miao Zhang, Lei Wang, Guofang Wu, Xing Wang, Haoxin Lv, Jun Chen, Yuan Liu, Huili Pang, Zhongfang Tan

**Affiliations:** ^1^Henan Key Laboratory of Ion-Beam Bioengineering, College of Agricultural Sciences, Zhengzhou University, Zhengzhou, China; ^2^Academy of Animal Science and Veterinary Medicine, Qinghai University, Xining, China; ^3^College of Food Science and Engineering, Henan University of Technology, Zhengzhou, China

**Keywords:** freezing and thawing temperatures, fermentation, lactic acid bacteria, silage, anti-bacteria

## Abstract

The corruption and/or poor quality of silages caused by low temperature and freeze-thaw conditions makes it imperative to identify effective starters and low temperature silage fermentation technology that can assist the animal feed industry and improve livestock productivity. The effect of *L. plantarum* QZ227 on the wheat silage quality was evaluated under conditions at constant low temperatures followed by repeated freezing and thawing at low temperatures. QZ227 became the predominant strain in 10 days and underwent a more intensive lactic acid bacteria fermentation than CK. QZ227 accumulated more lactic acid, but lower pH and ammonia nitrogen in the fermentation. During the repeated freezing and thawing process, the accumulated lactic acid in the silage fermented by QZ227 remained relatively stable. Relative to CK, QZ227 reduced the abundance of fungal pathogens in silage at a constant 5°C, including *Aspergillus*, *Sporidiobolaceae*, *Hypocreaceae*, *Pleosporales*, *Cutaneotrichosporon*, *Alternaria*, and *Cystobasidiomycetes.* Under varying low temperature conditions from days 40 to days 60, QZ227 reduced the pathogenic abundance of fungi such as *Pichia*, *Aspergillus*, *Agaricales*, and *Plectosphaerella*. QZ227 also reduced the pathogenic abundance of *Mucoromycota* after the silage had been exposed to oxygen. In conclusion, QZ227 can be used as a silage additive in the fermentation process at both constant and variable low temperatures to ensure fast and vigorous fermentation because it promotes the rapid accumulation of lactic acid, and reduces pH values and aerobic corruption compared to the CK.

## Introduction

The Qinghai-Tibet Plateau is the coldest region outside the poles, with an average elevation of more than 4,000 m (Qiu, 2008). Livestock production has always been the main economic activity in the alpine regions, but the alpine grassland on the plateau is very susceptible and vulnerable to climate change and human interference. Low and insufficient feed quantity are the main limiting constraints of livestock productivity and feed source utilization in alpine area (Alagawany et al., 2020). Natural pasture biomass reaches a maximum in early autumn and then gradually decreases during the cold season. This means that a large number of livestock are slaughtered before winter due to feed shortages. Therefore, the construction of a sustainable livestock production system for the continuous feeding of ruminants throughout the year is a particular requirement in alpine regions (Alonso et al., 2013).

Besides, the outer layer of the silage stacks is subject to temperature changes during fermentation, and oxygen touch caused by defective tightness. The risk of exposure to an aerobic environment may occur each time material is removed from the silo. This means that the outer layer of the silage stacks may be subject to corruption or the material is poor quality because of insufficient fermentation or secondary fermentation. When the silage silo is opened, the anaerobic environment becomes an aerobic environment and the microorganisms that hibernate during oxygen deficiency begin to proliferate, resulting in spoilage of the silage, commonly known as secondary fermentation. This process is characterized by rising temperatures and fungal growth, and leads to dry matter and nutrient losses.

Ensiling is an effective pretreatment technology for harvested crops. It creates an acidic environment that reduces the risk of feedstock decay and combustion under anaerobic conditions. However, the storage temperature is a critical parameter that significantly affects the silage quality by influencing the microbial community diversity in silages (Wang Y. et al., 2019; Ren et al., 2020). In addition, silage fermentation is limited by repeated freezing and thawing low temperatures because not enough lactic acid is produced to ensure the production of good quality silage (Cao et al., 2011; Zhou et al., 2016; Dong et al., 2019; Wang C. et al., 2019; Ren et al., 2020). One of the key solutions to silage corruption mentioned above in laboratory scale is to simulate the seasonal and outer-layer changing conditions of repeated freezing and thawing low temperatures.

The starter culture is another key to the fermentation quality of silage. Meanwhile, the hypoxia, reduced pressure, low temperatures, strong ultraviolet radiation, and other special climate conditions, along with the unique ecosystem environment of the Qinghai-Tibet Plateau, have led to unique resources of lactic acid bacteria (LAB) with strong stress tolerance abilities (Yang et al., 2014). In our previous study, more than 2,000 strains of LAB were isolated from plants, saline ± alkali soil, a brine lake, indigenous yogurt, and the intestines of *Gymnocypris przewalskii* from the Qinghai-Tibet Plateau at an altitude of 3,100 to 4,800 m (Li, 2013; Lv, 2014; Zhang et al., 2015; Zhang, 2018). Some of these samples showed excellent potential as silage inoculants (Roier et al., 2015; Zhang et al., 2017b, 2018b), and have considerable application potential in solving the silage corruption caused by insufficient fermentation or repeated freeze-thaw conditions. Freezing damage to forages represents a major economic loss to agriculture. Studies have shown that freeze-thaw treatment could promote the development of *Lactobacillus* during ensiling. Therefore, the control of aerobe revitalization needs to concentrate on silages made from freeze-damaged forages (Dong et al., 2019). In this study, we used a well proven strain, QZ227, to improve the fermentation quality of silage at low temperature and then evaluated the silage quality variations under low temperatures freezing and thawing. We simulated constant low temperature fermentation and then outer layer fermentation conditions under varying low temperatures. The QZ227 strain, isolated from the Qinghai-Tibet Plateau, was selected and the aim was to try and improve silage quality by first subjecting it to a low constant temperature of 5°C followed by repeated freezing and thawing at various low temperatures. We aimed to slow down the outer-layer silage losses and seasonal silage corruption caused by insufficient fermentation at low temperatures or secondary fermentation.

## Materials and Methods

### Characteristics of the Inoculum

The sources of the QZ227 inoculates are shown in Supplementary Table 1. The QZ227 strains were isolated from a wheat landrace grown on the Qinghai-Tibet Plateau at 3,100 m above sea level. Permission to use the sampling location was issued by Zhengzhou University, China, and local farmers. The QZ227 isolates were identified as *Lactobacillus plantarum* using 16S rRNA-based phylogeny combined with an assessment of their typical morphophysiological characteristics. Absorbance values at 560 nm and the pH values of the bacteria solution were measured every 2 h to determine its growth curve and acid-producing ability. The abilities of the isolates to resist NaCl, bile, acid, alkali, and low and higher temperature stress were measured through culturing in de Man, Rogosa, and Sharpe (MRS) broth containing 3.0 and 6.5% (w/v) NaCl or 0.1 and 0.3% (w/v) bile, with pH values from 1 to 10, at temperatures of 5 – 50°C, severally (Zhang et al., 2018a). The bacterial growth rates were assayed using the turbidimetry method at 550 nm combined with visual turbidity. The antimicrobial characters of QZ227 were evaluated by the method of bidirectional diffusion method (Balouiri et al., 2016), and the standard bacterial strains including *Micrococcus luteus* (*M. luteus*), *Escherichia coli* (*E. coli)*, *Staphylococcus aureus* (*S. aureus*), *Salmonella enterica* (*S. enterica*), *Listeria monocytogenes* (*L. monocytogenes*), *Pseudomonas aeruginosa* (*P. aeruginosa*), and *Bacillus subtilis* (*B. subtilis*) were bought from the China General Microbiological Culture Collection Center (CGMCC). The inhibition zone diameter was measured after anaerobic incubation at 30°C for 24 h.

### Inoculates and Silage Preparation

The whole profile for silage making and fermentation is shown in Supplementary Figure 1. Revitalized QZ227 isolates were inoculated into liquid MRS broth and cultured for 24 h. The viable cell concentration of QZ227 was 8 log CFU/mL.

Common hexaploid wheat plants grown in experimental field of Henan Provincial Key Laboratory of Ion Beam Bioengineering, Zhengzhou University, Xinxiang, China, was harvested on May 16, 2019 and sent to the silage laboratory within 2 h. The raw wheat materials were wilted for 10 h and then chopped into 2–3 cm lengths using a forage chopper. A total of 12 kg of chopped wheat and 800 mL of bacteria solution were thoroughly mixed, 800 g of the mixed material was placed in a plastic film bag (Dragon N-6; Asahi Kasei Co., Tokyo, Japan) degassed, and sealed by a vacuum sealer (SQ-203S Vacuum Sealer; Asahi Kasei Co., Tokyo, Japan). The packed silage bags were fermented in a refrigerator at 5°C for 30 days, afterward the fermentation condition changed to a variational temperature of 10°C during the day and −10°C during the night alternately. Three replicates of each treatment were opened every 10 days during the ensiling process. These were then used for the microbial and chemical analyses. The remaining samples were stored in a freezer at −80°C for further microbial genome sequencing.

### Viable Microbial Community

The dilution separation method was used to isolate and count the microbial community. A total of 10 g of silage was shaken with 90 mL of sterilized deionized water using a vortex mixer (Ika Vortex 3, Staufen, Germany), and serial dilutions (10^–1^ to 10^–5^) were prepared in sterilized filtered water. Then 20 μL 10^–1^, 10^–2^, and 10^–3^ dilutions were separately coated onto agar medium plates.

Different cultural media were used for various viable cell counts. Lactic acid bacteria were cultured in MRS medium at 30°C for 2 days anaerobically and then numerated. In the same way, NA culture was used for aerobic bacteria, the potato dextrose agar (PDA) contained a sterilized 10% dihydroxysuccinic acid solution (final concentration: 1.5%) was used for yeasts and molds. The *E. coli* were numbered using eosin methylene blue (EMB). The NA, PDA, and EMB agar media were purchased from QingDao Hope Bio-Technology Co., Ltd., Qingdao, China. The agar plates coated with the different dilutions were incubated at 30°C for 2 days. Then, the 10^–1^ and 10^–2^ dilutions were heated in a constant water bath at 75°C for 15 min and coated onto NA and *Clostridium difficile* agar so that the *B. subtilis* and *C. difficile* colonies, respectively, could be counted. All the colonies were counted and the logarithmic number of viable colony-forming units in fresh matter (l g CFU/g FM) was calculated.

### Microbial Community Composition

A total of 10 g of refrigerated silage in 40 mL sterile deionized water was vibrated by an electron oscillator at 120 rpm for 2 h and then filtered using double gauze masks. A total of 40 mL sterile water was used to wash the microbial residues off the gauze. The filtrate was centrifuged for 15 min at 10,000 × *g*∼12,000 × *g* and 4°C, and the solids were collected and stored at −80°C for further research.

The Metagenomics Sequencing was commissioned to Majorbio Bio-Pharm Technology Co., Ltd. (Shanghai, China). Follows the steps (Jiang et al., 2019; Yang et al., 2019): (i) DNA extraction and PCR amplification, The kit we selected is E.Z.N.A. Soil DNA Kit purchased from Omega Bio-tek (Norcross, GA, United States). (ii) Illumina MiSeq sequencing, amplicon sequencing was conducted using an Illumina MiSeqPE250 platform (Shanghai Majorbio162 Bio-pharm Technology Co., Ltd., China). (iii) Processing of the sequencing data, the original 16S rRNA sequencing reads were demultiplexed and quality-filtered by the software of Trimmomatic^1^, and merged by FLASH based on the indexes below: (i) the 300 bp reads were truncated receiving an average quality score of < 20 over a 50 bp sliding window. (ii) Only overlapping sequences longer than 10 bp were assembled according to their overlapped sequence. The maximum mismatch ratio for the overlap region was 0.2. Reads that could not be assembled were discarded. (iii) Samples were distinguished according to their barcode and primers, and the sequence direction was adjusted based on exact barcode matching. A 2 nucleotide mismatch was allowed during primer matching. Operational taxonomic units (OTUs) with a 97% similarity cutoff (Liu et al., 2017) were clustered using UPARSE (version 7.1^2^), and chimeric sequences were identified and removed. The taxonomy of each OTU representative sequence was analyzed by RDP Classifier^3^ against the 16S rRNA database (e.g., Silva SSU128) using a confidence threshold of 0.7.

### Chemical Composition

A total of 10 g of silage materials were mixed with 90 mL of sterilized, purified water. About 50 mL of the liquid, after the organisms and visible solid matters had been filtered out with qualitative filter paper, was stored in a sterile container at −20°C for further NH_3_-N and organic acid analyses (Shah et al., 2020a,b), while the remaining mixture was used to determine the pH. The dry matter, crude protein, and crude ash were assayed by the atmospheric pressure drying method (AOAC 934.01), the high temperature ashing method (AOAC 942.05), and the Kjeldahl method (AOAC 979.09), respectively.

Silage remained was further dried in an thermostatic blast furnace (Shanghai Shilu Instrument Co., Ltd., Shanghai, China) at 65°C for 2 days and then the percentage dry matter and free water contents were calculated according to the following formulas:

$$\mathrm{Dry}\mathrm{matter}\left(\%\mathrm{FM}\right)=\frac{\left(\mathrm{M2}-\mathrm{M0}\right)*100\%}{\mathrm{M1}-\mathrm{M0}}$$

*Freewater*(%*FM*)100%-DM%

M0, mass of the silage bag

M1, mass of silage prior to drying

M2, mass of the silage after it had been dried for 48 h.

The dried silage was ground using a high speed pulverizer (FW-100, Taisite Instrument Co., Ltd., Jinghai, Tianjin, China). The crude protein content of the silage powder was determined using automatic Kjeldahl apparatus (K1100, Jinan Hanon Instruments Co., Ltd., Jinan, China) with CuSO_4_ and K_2_SO_4_ at a ratio of 1:15 as the catalyst. The standard concentration of the titrated H2SO4 solution was determined by color indicator titration method (Zhang et al., 2018b) with anhydrous sodium carbonate and bromocresol green ± methyl red as an color indicator. The crude ash content was calculated after incineration for 2 h at 550°C in a muffle furnace (REX-C900, RKC Instrument Inc., Osaka, Japan), followed by burning in an ashing furnace until no smoke was produced. The detergent fiber was determined by the Automatic Fiber Determination System (CXC-06, Zhejiang Top Instrument Ltd., Hangzhou, China). The organic acid content in silage were measured by high performance liquid chromatography, the chromatographic conditions and sample pretreatment methods were established according to the related literatures (Ni et al., 2017; Zhang et al., 2018b), the standard acids were purchased from Sigma-Aldrich Chemical Co. (St. Louis, MO, United States). The NH_3_-N was measured using the indophenol blue method as described. A standard formula was created using an NH_4_Cl solution as the reference. The absorbance of the sample was compared with the standard formula to determine the NH_4_Cl content using the following equation:

$${\text{NH}}_{3}-N\,\left(g/\mathrm{kg}\,\mathrm{dry}\,\mathrm{matter}\right)=\frac{\left(N\,{\text{H}}_{4}\,\mathrm{Cl}\,\mathrm{concentration}\right)*17}{53.5*\left(\mathrm{silage}\,\mathrm{dry}\,\mathrm{matter}\right)}.$$

### Statistical Analyses

Two-way analysis of variance was conducted with inoculation amount and fermentation time as the primary variables. Many variables interact significantly with each other, so the inoculation effect at each fermentation time was examined by one-way analysis of variance (ANOVA), followed by a multiple comparison by Tukey’s test. The statistical significance of the antibacterial activity and the NH_3_-N producing ability of pathogens were evaluated using ANOVA with Tukey’s HSD test. These analyses were carried out using IBM SPSS Statistics 19 software^4^, and the pH variations and microbial communities were plotted using Origin software Pro 8.5.1^5^. A *P*-value of < 0.05 was considered statistically significant and a *P*-value of < 0.01 was considered very significant. The bacterial and fungal sequencing data were analyzed using the free online Majorbio Cloud Platform^6^.

## Results and Discussion

### Characteristics of the Inoculum

The morphological characteristics, physiological and biochemical properties of QZ227 are shown in Supplementary Table 1. QZ227 cannot use glucose to produce gas, which means that it has a fermentation type called homofermentation. It is characterized as being low temperature resistant and has good high temperature tolerance because it can grow well at 5 and 50°C. Furthermore, it can grow well at 3.0 and 6.5% NaCl and under 0.1 and 0.3% bile conditions. Therefore, the QZ227 isolates were tolerant of bile and salt. In addition, QZ227 can live in extremely acid conditions of pH 2.0 and extremely alkaline conditions of pH 10.0.

The growth curve determination process showed that Q227 produced lactic acid and acetic acid (Supplementary Figures 1, 2). After cultivation for 48 h, the lactic acid and acetic acid concentrations in QZ227 were 44.99 and 8.85 mg/mL, respectively.

Table 1 shows that QZ227 has broad-spectrum antibacterial activity and an antibacterial effect on gram-negative pathogens, including *Escherichia coli, Pseudomonas aeruginosa, Salmonella enterica*, and Gram-positive pathogens, including *Micrococcus luteus* and *Staphylococcus aureus*.

**TABLE 1:** The pathogen inhibition of QZ227.

	*Micrococcus luteus*	*Staphylococcus aureus*	*Bacillus subtilis*	*Escherichia coli*	*Pseudomonas aeruginosa*	*Salmonella enterica*
CK	−	−	−	−	−	−
QZ227	+++	+++	−	++	+++	+++

*The inhibition zone contains the external diameter of oxford cup (7.68 mm). Inhibition zone: Less than 8.00 mm, −; 8.00–12.00 mm,+; 12.00–16.00 mm,++; More than 16.00 mm,+++.*

### Silage Preparation

The chemical composition and microbial community composition of the raw materials prior to ensiling is shown in Supplementary Table 2. The pH of the raw materials was 6.57 and the moisture content was 63.48% FM. The crude protein and ether extract contents were 4.65 ± 0.12% DM and 12.51 ± 0.75% DM, respectively, and the neutral detergent fiber (NDF) and acid detergent fiber (ADF) contents were 50.37 ± 2.21% DM and 34.12 ± 1.55% DM, respectively. No organic acid was detected. The LAB counts for the raw materials were 2.69 ± 0.37 log CFU/g FM. *E. coli, F. fungi, S. cerevisiae*, and *B. subtilis* were detected, but aerobic bacteria and *S. marcescens* were not detected in the raw materials.

### Treatment Effects on pH

**FIGURE 1:**
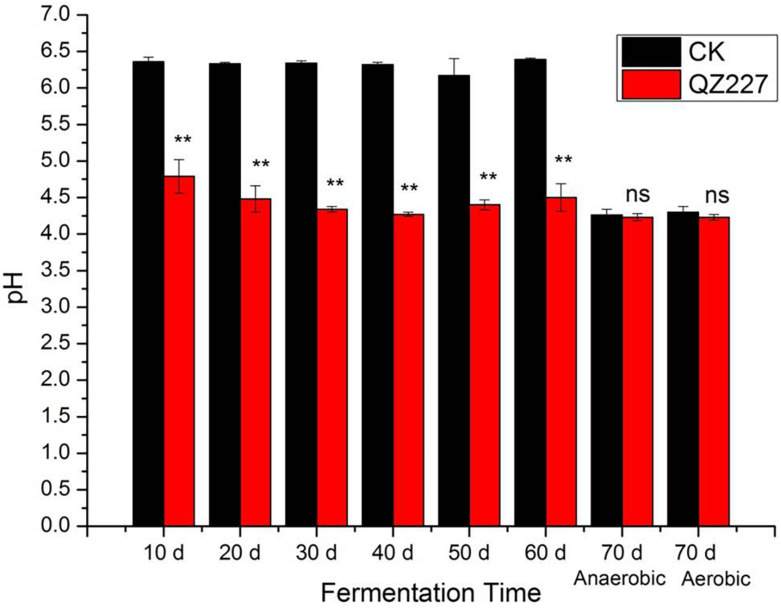
The pH variations during the fermentation process. Ns, not significant, **, very significant, 0.001 < *P* ≤ 0.01.

As shown in Figure 1, the pH of the silage inoculated with QZ227 had decreased to 4.27 by day 40. Furthermore, the pH of the silage inoculated with LAB remained relatively low in the low temperature environment. However, the pH of the CK group without inoculates was undesirably above 6.17 during the whole variable low temperature process where the temperature varied from −10 to 10°C. As the fermentation temperature rose to 25°C from day 60 to day 70, the pHs of all the silage groups decreased under both the anaerobic and aerobic conditions.

**FIGURE 2:**
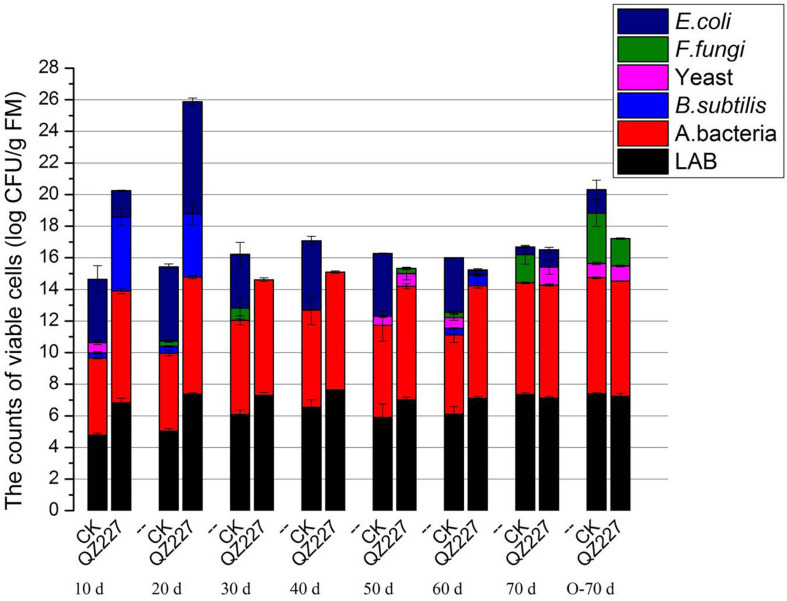
The number of cultural microorganisms during the fermentation process.

The pH is the main indicator of silage preservation quality and fermentation efficiency, and represents the final acidity of the fermentation product. The feeding intakes and rumen digestion stability of silage with low pH are poor, but a higher pH is often accompanied by a greater ammonia nitrogen content, which also leads to a poorer preservation quality and lower feed intake.

The dry weight contents should be combined when making a silage pH evaluation. A silage with a dry weight content of less than 28% has a higher quality when the pH is between 3.8 and 4.2, and silage with a dry weight greater than 28% can be better preserved with a pH greater than 4.5. Moist silage with a pH greater than 4.2 is difficult to preserve over long periods of time. In addition, when the pH exceeds 4.4 (except for low moisture silage), the activities of spoilage bacteria and butyric acid bacteria are generally more intense during the silage fermentation process. The dry matters of the silage in this study were above 33%, at the various low temperatures experienced from days 10 to 60. The pH of the CK group without inoculates indicated that it would be difficult to preserve the silage, whereas the pH of the inoculated silage suggested that QZ227 improved preservation.

During the silage process, lactic acid bacteria convert carbohydrates in raw materials into organic acids. The most important ingredient is lactic acid. The higher the lactic acid content, the better the aromatic flavor of the feed, the higher the feed intake, and the better the aerobic stability (Li et al., 2014). When the lactic acid content is 8–10% DM and it accounts for more than 75% of the total acid content, then the quality of the silage is good, but levels below 5% DM lead to poor quality silage. Many factors have been shown to affect the proportion of produced organic acids, including the microbial population, inoculum source, substrate complexity, nutrient availability, pH, and temperature (Bastidas-Oyanedel et al., 2015).

No lactic acid was detected in the CK group during the variant low temperature fermentation stage. During the continuous low temperature fermentation (5°C) stage, the lactic acid content of the silage inoculated with QZ227 continuously increased to 3.71 ± 1.31 mg/ml at day 30, but the lactic acid content of the fermented silage decreased to 2.04 ± 0.35 mg/ml when subjected to temperature variation. In this study, QZ227 promised a better aerobic stability than CK group.

The acetic acid contents of the QZ227 and the CK silages were not significantly different throughout the whole fermentation process. No propionic acid was detected during continuous low temperature fermentation and during the following variable temperatures stage. Propionic acid is produced in silage when the temperature rises to 25°C under both anaerobic and aerobic conditions. Heterofermentative lactic acid bacteria produce high concentrations of acetic or propionic acid. These acids inhibit the growth of spoilage yeasts and molds and improve the shelf life of the silage during feedout (Queiroz et al., 2013). However, they do not affect the relative abundances of predominant bacteria, such as *Lactobacillus*, *Weissella*, or *Pediococcus* (Ogunade et al., 2018). Propionic acid can be added as an antifungal agent to enhance the shelf life of silages during feedout (Kung et al., 2000). The propionic acid content of the QZ227 silage was higher than the CK group at 25°C although the increase was not significant (*P* ≥ 0.05).

The dry matter contents of the CK group fermented at low temperature from days 10 to 60 was 33.77–34.12% and the dry matter of the silage fermented with QZ227 was from 33.30 to 34.22%. The psychrotrophic *L. plantarum* inoculates lowered the moisture content of silage at low temperature compared with raw materials, which promised less DM loss during conservation (Zhang et al., 2017a). The results of this study were consistent with the phenomena. In this study of a constant low temperature of 5°C, the dry matter content of control group reduced to 33.77 ± 0.44% from 34.45 ± 0.14%, while the QZ227 group reduced to 33.30 ± 0.33 from 33.62 ± 0.20%. QZ227 promised less DM loss at the constant low temperature of 5°C, which were consistent with the research before (Zhang et al., 2018b).

### Cultural Microorganisms

During the natural silage process, the epiphytic lactic acid bacteria on the plant surface plays a decisive role in the quality of the final silage. Figure 2 shows that *F. fungi* and *E. coli* were detected in the CK group after the silage had fermented for 30 days, but neither were found in the QZ227 silage. At day 60, *S. cerevisiae*, *F. fungi*, and *E. coli* were found in the CK group, whereas they were not detected in the QZ227 group. These results, combined with the antibacterial activity (Supplementary Table 1), demonstrated that QZ227 had broad-spectrum antibacterial activity and can effectively inhibit the pathogens found in silage.

**FIGURE 3:**
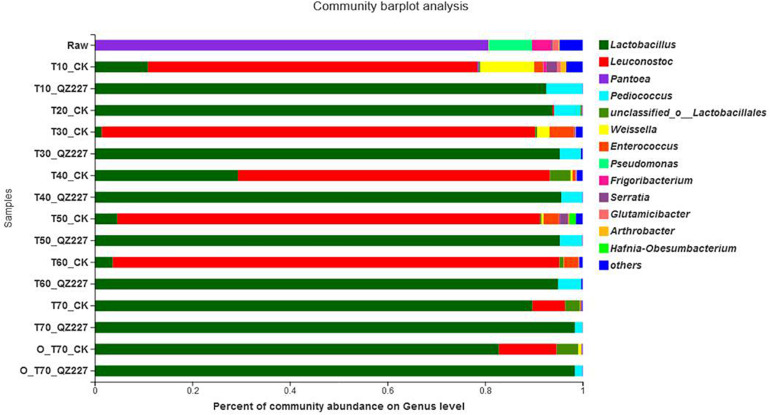
Analysis of bacterial community composition.

When the temperature increased to 25°C and the conditions were anaerobic for 10 days, pathogens *S. cerevisiae*, *F. fungi*, and *E. coli* occurred in QZ227, but the *F. fungi* and *E. coli* numbers were lower than in the CK group. Similar pathogen trends to anaerobic fermentation were detected when the temperature increased to 25°C and the silage was exposed to an aerobic environment for 10 days (Figure 3).

QZ227 can inhibit viable *F. fungi* and *E. coli* in silage exposed to aerobic conditions and atmospheric temperatures. However, under anaerobic conditions and atmospheric temperatures, the QZ227 only inhibited *F. fungi*.

The main factors affecting fermentation are fermentation time and inoculates. The principal effect analysis showed that the chemical components of the silage were significantly affected by fermentation time and inoculates. However, the NH_3_-N levels were significantly affected by the interactions between fermentation time and inoculates (Table 2).

**TABLE 2:** Variations in chemical components (% FM) and principal effect analysis.

Items	Additives	Fermentation time						Sig.			Fermentation time		Sig. at days 70		
					
		10 d	20 d	30 d	40 d	50 d	60 d	T	I	T × I	70 d	O-70 d	O	I	O × I
Lactate	CK	0.00 ± 0.00^*A*^	0.00 ± 0.00^*a*^	0.00 ± 0.00^*A*^	0.00 ± 0.00^*A*^	0.00 ± 0.00^*a*^	0.00 ± 0.00^*a*^	**	**	**	5.62 ± 0.66	2.93 ± 0.41	**	ns	**
	QZ227	1.85 ± 0.42^*B*^	3.00 ± 0.34^*b*^	3.71 ± 1.31^*B*^	3.85 ± 0.96^*B*^	2.61 ± 0.85^*b*^	2.04 ± 0.35^*b*^				4.45 ± 0.91	3.36 ± 0.85			
Acetic acid	CK	1.16 ± 0.3	1.41 ± 0.52	1.72 ± 0.32	2.41 ± 0.37	1.66 ± 0.48	1.62 ± 1.12	**	ns	ns	1.05 ± 0.12	0.65 ± 0.46	ns	ns	ns
	QZ227	0.97 ± 0.17	1.42 ± 0.09	1.79 ± 0.61	1.64 ± 0.56	1.23 ± 0.38	0.95 ± 0.25				1.37 ± 1.03	1.22 ± 0.91			
Propionic acid	CK	nd	nd	nd	nd	nd	nd	**	ns	**	2.06 ± 013	0.91 ± 0.79	*	ns	*
	QZ227	nd	nd	nd	nd	nd	nd				2.17 ± 0.52	1.59 ± 0.16			
NH_3_-N	CK	0.18 ± 0.03	0.16 ± 0.04	0.24 ± 0.01	0.34 ± 0.04	0.37 ± 0.06	0.33 ± 0.05^*a*^	**	**	*	0.47 ± 0.05^*a*^	0.35 ± 0.02	**	**	ns
	QZ227	0.21 ± 0.03	0.08 ± 0.08	0.16 ± 0.08	0.31 ± 0.07	0.29 ± 0.05	0.22 ± 0.05^*b*^				0.34 ± 0.05^*b*^	0.31 ± 0.03			
Dry matter	CK	34.45 ± 0.14^*A*^	34.59 ± 0.21	33.77 ± 0.44	34.28 ± 0.41	34.12 ± 0.52	34.24 ± 0.12^*a*^	**	**	ns	33.55 ± 0.34	33.36 ± 0.05	ns	ns	**
	QZ227	33.62 ± 0.20^*B*^	34.09 ± 0.42	33.30 ± 0.33	34.03 ± 0.14	34.22 ± 0.98	33.72 ± 0.26^*b*^				33.49 ± 0.01	33.03 ± 0.21			

No butyric acid were detected. Sig. Significance, T Time, I Inoculates, O Oxygen. Ns, not significant, *0.01 < *P* ≤ 0.05, **P ≤ 0.01. ^*ab*^ different letters indicate significantly differences at 0.01 < P < 0.05; ^*AB*^ different letters indicate significantly differences at P ≤ 0.01.

The organic acid levels were significantly affected by fermentation time. Lactic acid was significantly affected by inoculates, but acetic acid and propionic acid were not. Lactic acid was also significantly affected by the interactions between time and inoculates.

The cultured microorganisms, except *S. cerevisiae* and *F. fungi*, were also significantly affected by fermentation time and inoculates. The principal factors at day 70 were oxygen and inoculates. Table 2 shows that oxygen had a significant effect on NH_3_-N and lactic acid, whereas inoculates had a significant effect on both pH and NH_3_-N.

### Microbial Community Composition

The most abundant epiphytic bacteria in the raw wheat were *Pantoea*, followed by *Pseudomonas*. In the first 30 days of low temperature fermentation and the following repeated freeze-thaw stage, *Leuconostoc* was the predominant strain in CK while *Lactobacillus* was the predominant strain in silage inoculated with QZ227. The inoculants boosted the dominant fermentation flora and changed the bacterial composition. *Lactobacillus* abundance increased quickly in CK after 10 days of high temperature storage.

The bacterial community heatmap at the genus level is shown in Supplementary Figure 4. They showed that the bacterial community in the silage was affected by inoculates, and the different bacterial categories were differentially affected by the inoculates factors.

**FIGURE 4:**
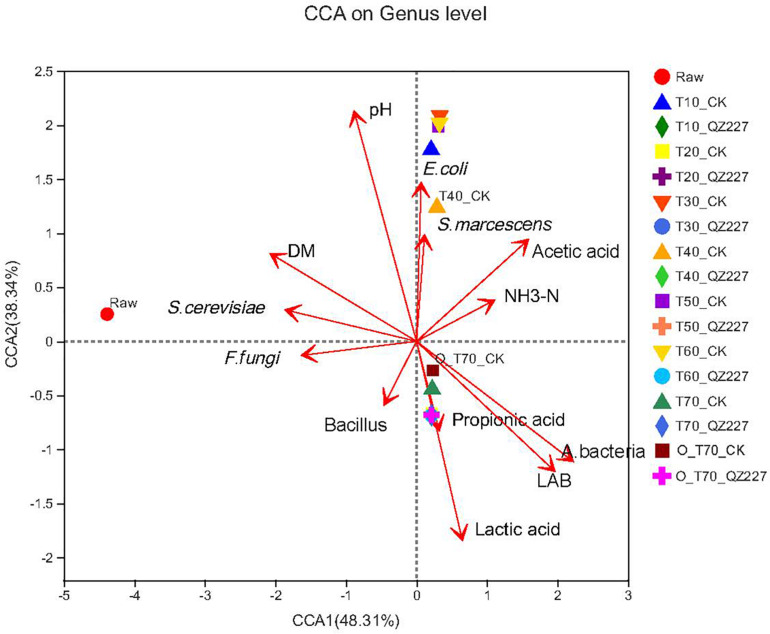
Bacteria canonical correlation analysis (CCA) at the genus level. The dots in different colors or shapes represent the environment or condition sample groups. The red arrows represent quantitative environmental factors, and the length of the arrows for environmental factors can represent the degree of influence (explanatory volume) that an environmental factor has on the species data. The distance from the projection point of the samples to the origin represents the relative influence of the environmental factor on the distribution of the sample community.

The results showed that pH had a negative correlation with lactic acid and viable lactic acid bacteria, but had a positive correlation with viable *E. coli, S. marcescens, S. cerevisiae, F. fungi*, and DM (Figure 4). Under aerobic conditions for 70 days, the lactic acid, propionic acid, lactic acid bacteria, and aerobic bacteria had greater impacts on the silage fermented with QZ227 than on the CK group. Similarly, under anaerobic conditions for 70 days, lactic acid, propionic acid, lactic acid bacteria, and aerobic bacteria also had greater impacts on QZ227 silage than on the CK silage.

**FIGURE 5:**
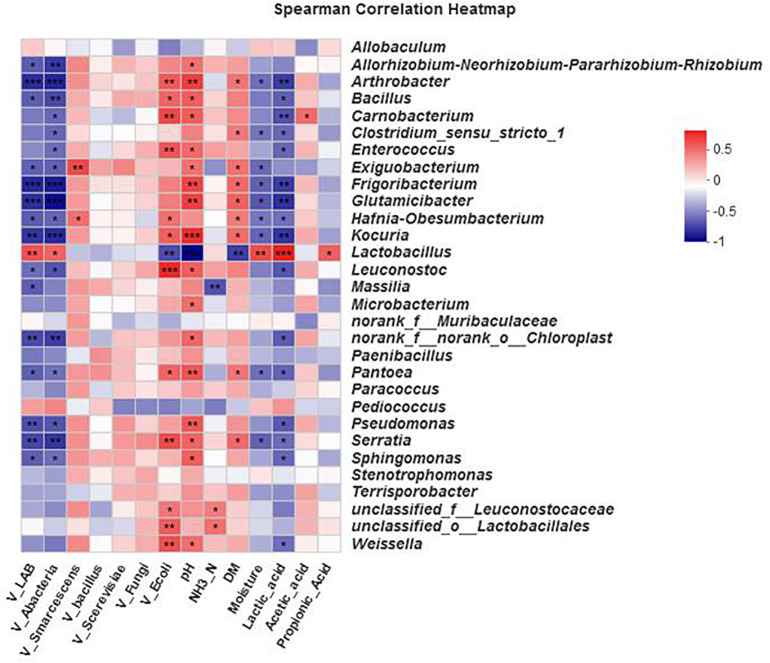
Spearman’s correlation heat map for bacteria at the genus level. ^∗^0.01 < *P* ≤ 0.05, ^∗∗^0.001 < *P* ≤ 0.01, ^∗∗∗^*P* ≤ 0.001. Microbial classification and environmental variables. The *x*-axis and *y*-axis are environmental factors and species, respectively. Different *R* values are shown in different colors.

The effects of different environmental factors on bacterial community composition in the silage are shown in the Spearman correlation heat map (Figure 5). The bacterial community compositions in the silage were affected by viable lactic acid bacteria, aerobic bacteria, *E. coli*, pH, DM, moisture, and lactic acid. The bacterial genera were differentially affected by the environmental factors. The lactobacillus genus was also significantly affected by propionic acid content (0.01 < *P* ≤ 0.05).

**FIGURE 6:**
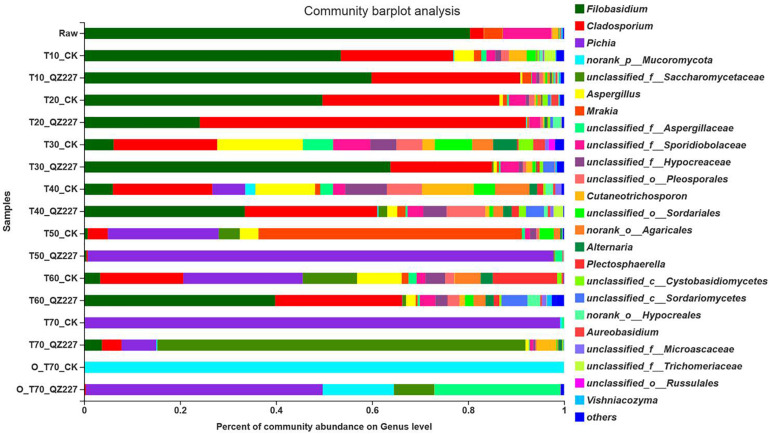
Fungal community barplot analysis at the genus level. The *y* – axis is the name of the sample, the *x* – axis is the proportion of the species, Colored columns indicate different genera, and the length of the columns represents the proportion of the genera.

According to Figure 6, *Filobasidium* was the most abundant fungal community in the raw materials and silage fermented for 10 days. At day 30, more fungal genera were detected in the CK group than in QZ227, and the percentages for pathogens in the CK group, such as *Aspergillus*, *Sporidiobolaceae*, *Hypocreaceae*, *Pleosporales*, *Cutaneotrichosporon*, *Alternaria*, and *Cystobasidiomycetes*, were larger than in QZ227. We concluded that QZ227 decreased the growth of these fungal pathogens in silage under low temperature (5°C).

From day 40 to day 60 of varying low temperature conditions, the main fungal genus in QZ227 was more obvious and uniform than in the CK group. The numbers and varieties of species from the main fungal genus in the CK group were not clear. QZ227 inhibited the growth of certain fungi genera from days 30 to 60. At day 40, the fungal pathogen percentages for *Pichia*, *Mucoromycota*, *Aspergillus*, *Hypocreaceae*, *Sordariales*, and *Agaricales* in CK were higher than in QZ227. At day 50, the fungal pathogen percentages for *Cladosporium*, *Saccharomycetaceae*, *Aspergillus*, *Mrakia*, *Sporidiobolaceae*, *Hypocreaceae*, *Pleosporales*, and *Sordariales* in CK were higher than in QZ227. At day 60, the *Pichia*, *Alternaria*, *Aspergillus*, *Agaricales*, *Plectosphaerella* percentages were higher than in QZ227. We concluded that QZ227 inhibited fungal growth under varying low temperature conditions.

Antifungal supplements are mostly used to hinder the growth of yeasts, fungi, and other undesirable microorganisms to improve fermentation of the silage (Shah et al., 2020b). Yeast has long been considered to be the main microorganism that causes aerobic deterioration in silage because the aerobic deterioration of silage is closely related to the metabolism of the main yeast strain (da Silva et al., 2020). The yeasts that cause aerobic deterioration in silage were divided into two groups. The first contained acid-using fungi, such as *Candida*, *Endomycopsis*, *Hansenula*, and *Pichia*. The other group used sugars, such as the genus *Torulopsis*, *Pichia manshurica*, *Candida ethanolica*, and *Zygosaccharomyces bailii*, which meant that they were able to resist acetic acid and, therefore, had a greater effect on the aerobic metamorphism of silage during the early fermentation stage (Wang et al., 2018). At the later stage of silage production, *Zygosaccharomyces bailii* was the main yeast causing aerobic metamorphism in silage.

Figure 6 shows that at day 70, the dominant fungi in CK was *Pichia*, which meant that the CK group silage was in the initial stage of aerobic decay at day 70.

*Mucoromycota*, *Aspergillus bubble*, *Rhizopus oryzae*, *Penicillium acanthopanax*, and *Penicillium decumbens* were detected in moldy silage (Fu, 2012). In this study, after exposure to oxygen for 70 days, *Mucoromycota* were detected in the CK group, which meant that the CK group became moldy after exposure to oxygen for 70 days. *Pichia* were the dominant fungi while the *Mucoromycota* proportion was small in silage fermented with QZ227, which suggested that QZ227 inhibited the growth of *Mucoromycota*, and slowed down the speed and extent of corruption when the silage was exposed to oxygen.

**FIGURE 7:**
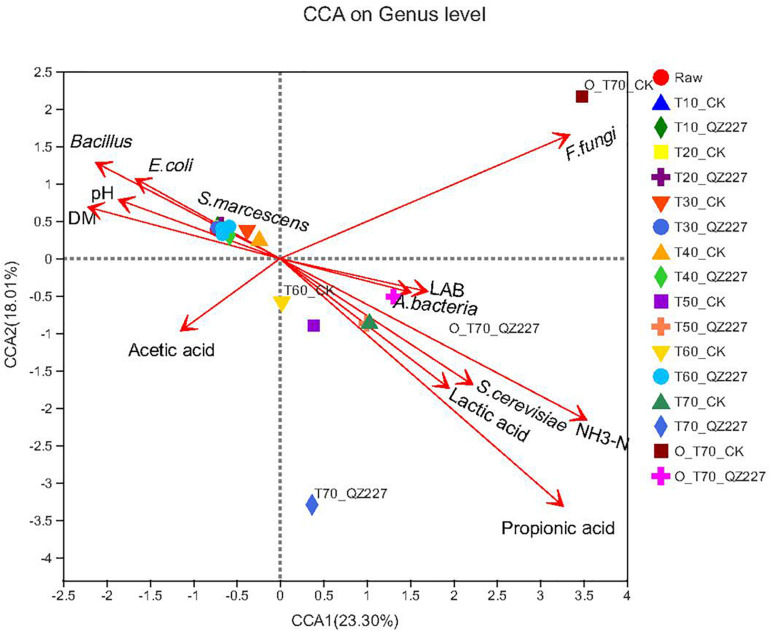
Fungi CCA at the genus level. The dots in different colors or shapes represent the environment or condition sample groups.

Figure 7 shows that pH had a negative correlation with lactic acid, and viable lactic acid bacteria had a positive correlation with viable *E. coli*, *S. marcescens*, and DM, which was consistent with Figure 4. Under aerobic conditions for 70 days, the lactic acid and propionic acid had a greater impact on the silage fermented with QZ227 compared to the CK group, which was consistent with Figure 4. Similarly, under anaerobic conditions for 70 days, the lactic acid, propionic acid, lactic acid bacteria, and aerobic bacteria had a greater impact on QZ227 compared to CK, which was also consistent with Figure 4.

**FIGURE 8:**
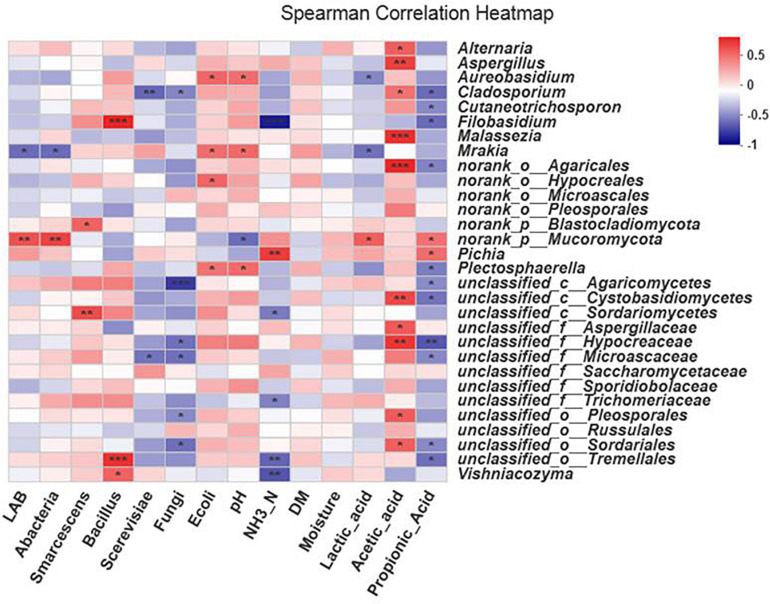
Spearman’s correlation heat map for fungi at the genus level. ^∗^0.01 < *P* ≤ 0.05, ^∗∗^0.001 < *P* ≤ 0.01, ^∗∗∗^*P* ≤ 0.001. The *x*-axis and *y*-axis are environmental factors and species, respectively. Different *R* values are shown in different colors.

The fungal community composition in the silage was affected by all the environmental factors except DM and moisture, which had no effect on fungal community composition (Figure 8).

### Intragroup Difference Analysis of Paralleled QZ227 at Day 60

The pHs of the three paralleled silages in the QZ227 group were 4.49, 4.70, and 4.32, respectively, and these differences were relatively large. The bacterial and fungal species in the paralleled QZ227 silages at day 60 were then analyzed.

**FIGURE 9:**
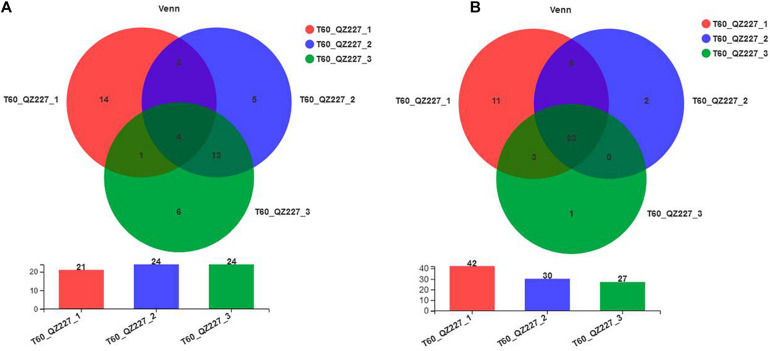
Bacterial **(A)** and fungal **(B)** species in the paralleled QZ227 groups at day 60. The numbers in the overlapped areas represent the number of genera common to multiple groups and the numbers in the non-overlapped areas represent the number of genera unique to the corresponding group.

Figure 9A shows that there were few or no differences between the number of bacterial genera among intro-groups QZ2271⃝, QZ2272⃝, and QZ2273⃝. However, each of them contained a unique genus of bacteria. The fungal genera among the QZ227 intro-groups were different (Figure 9B). The number and varieties of microorganisms probably affected the pH of the QZ227 intro-groups.

**FIGURE 10:**
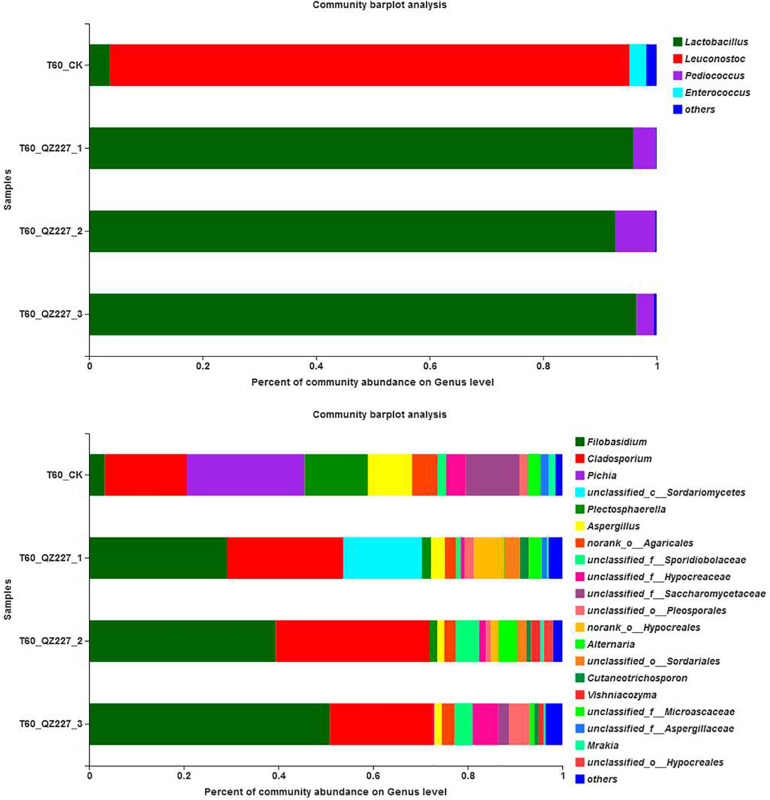
Community abundances of the paralleled QZ227 groups at day 60.

Figure 10 shows that *Lactobacillus* was the predominant stain. However, *Pediococcus* were also detected and *Pediococcus* abundance increased with pH in QZ227. The fungal community abundances were different among the QZ227 intro-groups.

Above all, the pHs of the QZ227 introgroups were significantly different. Possible reasons for these differences were the different number and varieties of microorganisms, and the different *Pediococcus* and fungal community abundances. The bacterial and fungal numbers, varieties, and abundances were different, even when they were treated with same inoculates and conditions. However, the pHs of the silage inoculated with QZ227 were lower and this would improve their storage potential compared to the CK group.

## Conclusion

QZ227 has good stress tolerance of temperature, bile, salt, acid, and alkali. It has a strong acid-producing capacity and broad-spectrum antibacterial activity, and can effectively inhibit several pathogens that occur in silage. The inoculant boosted the dominant fermentation flora and changed the bacterial composition after 30 days. The pH of the silage inoculated with QZ227 decreased to 4.27 at day 40 and remained at a relatively low level in the subsequent variable low temperature stage, which will improve storage. In both the constant low temperature and variable low temperature fermentation stages, lactic acid was detected in silage inoculated with QZ227, but not in CK. The results for the high-throughput sequencing combined with viable cell culture counting method showed that QZ227 became the predominant strain and inhibited the fungal growth of *Aspergillus*, *Sporidiobolaceae*, *Hypocreaceae*, *Pleosporales*, *Cutaneotrichosporon*, *Alternaria*, and *Cystobasidiomycetes* at a constant low temperature of 5°C. Under the variable temperature conditions from days 30 to 60, QZ227 reduced the fungal pathogens percentages of *Pichia*, *Mucoromycota*, *Aspergillus*, *Hypocreaceae*, *Sordariales*, *Agaricales*, *Cladosporium*, *Saccharomycetaceae*, *Mrakia*, *Sporidiobolaceae*, *Pleosporales*, *Alternaria*, and *Plectosphaerella.* QZ227 also inhibited the growth of *Mucoromycota* and slowed down the speed and extent of corruption when the silage was exposed to oxygen. The results showed that QZ227 has the potential to be used as a starter culture for wheat fermentation at low temperature and as a protective spray for the outside layers of silage stacks.

## Data Availability Statement

The raw data supporting the conclusions of this article will be made available by the authors, without undue reservation.

## Author Contributions

ZT: conceptualization. MZ: data curation, software, and writing – original draft. ZT and MZ: formal analysis, investigation, writing, review, and editing. ZT, LW, and GW: funding acquisition. XW, HL, JC, and YL: resources. HP and ZT: supervision. All authors contributed to the article and approved the submitted version.

## Conflict of Interest

The authors declare that the research was conducted in the absence of any commercial or financial relationships that could be construed as a potential conflict of interest.

## Footnotes

1 http://www.usadellab.org/cms/index.php?page=trimmomatic

2 http://drive5.com/uparse/

3 http://rdp.cme.msu.edu/

4 https://www.ibm.com/analytics/cn

5 http://www.originlab.com/index.aspx?go=Products/Origin

6 www.majorbio.com
