# Supplementary figures and images for: Effects of Lactobacillus plantarum on the Fermentation Profile and Microbiological Composition of Wheat Fermented Silage Under the Freezing and Thawing Low Temperatures
Source: Front Microbiol. 2021 Jun 9;12:671287. doi: 10.3389/fmicb.2021.671287 (PMC8221580; doi:10.3389/fmicb.2021.671287)

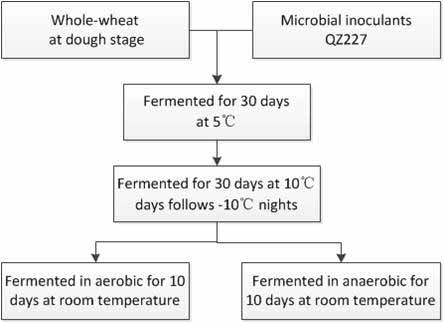

Supplement: Supplementary file 1 [file Image_1.JPEG]

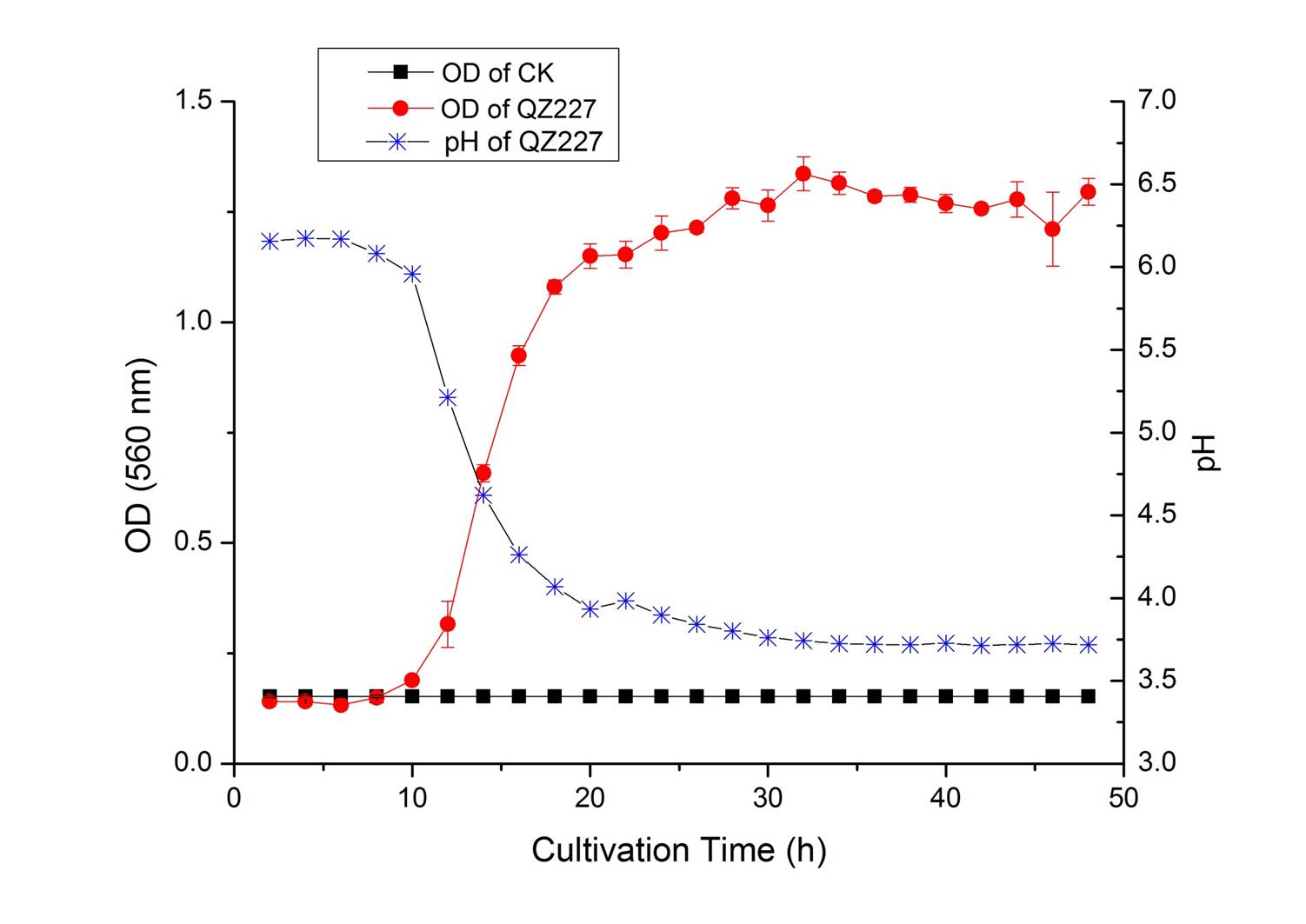

Supplement: Supplementary file 2 [file Image_2.JPEG]

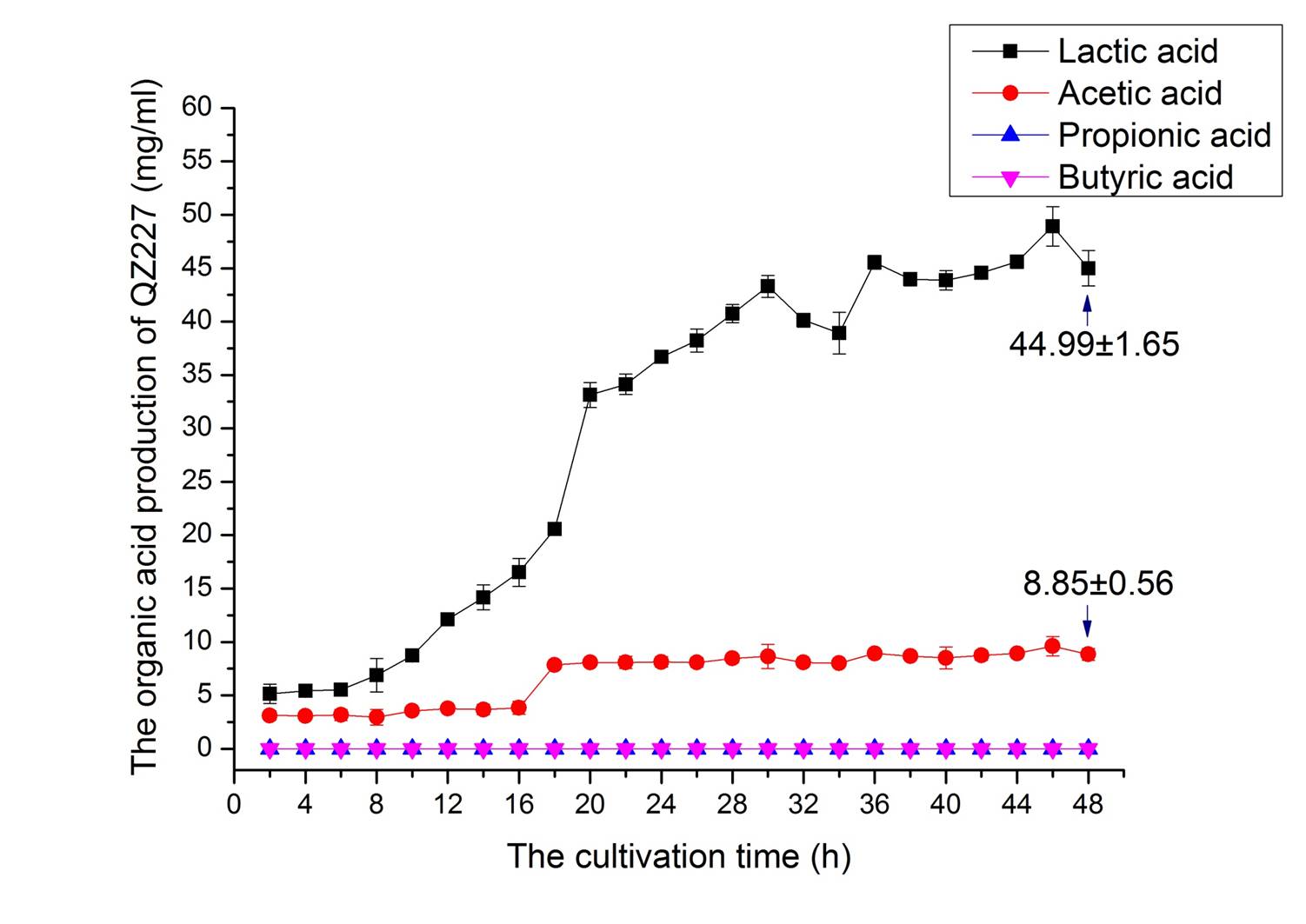

Supplement: Supplementary file 3 [file Image_3.JPEG]

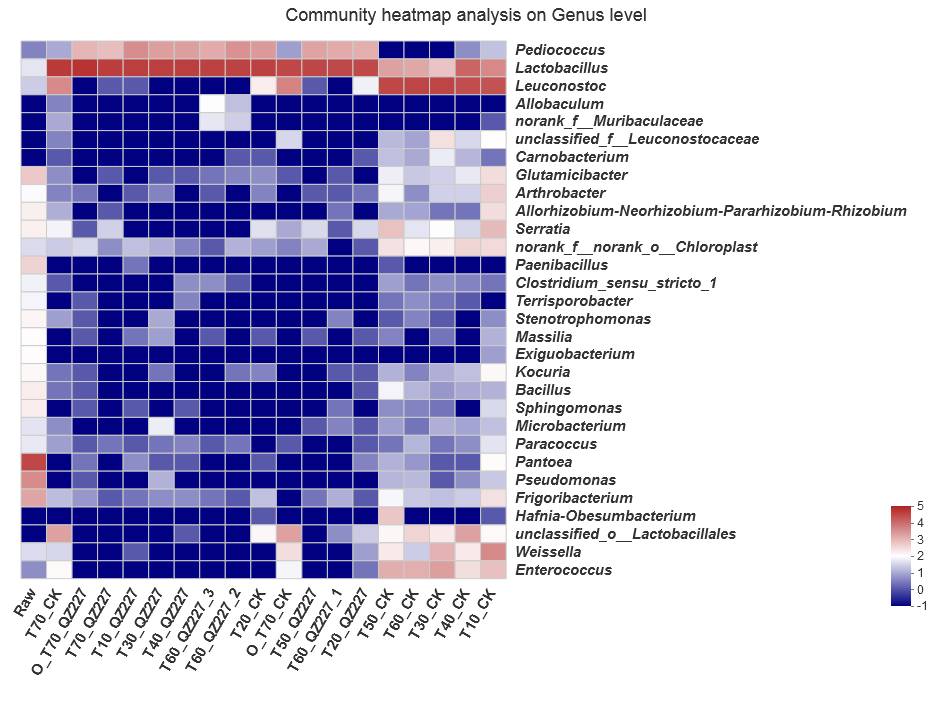

Supplement: Supplementary file 4 [file Image_4.JPEG]
