# Supplementary material for: Effects of Lactobacillus plantarum on the Fermentation Profile and Microbiological Composition of Wheat Fermented Silage Under the Freezing and Thawing Low Temperatures
Source: Front Microbiol. 2021 Jun 9;12:671287. doi: 10.3389/fmicb.2021.671287 (PMC8221580; doi:10.3389/fmicb.2021.671287)
Supplement: Supplementary file 5 [file Table_1.DOCX]

**Supporting Tables**

**Supporting Table S1**. Morphological, physiological and biochemical properties of inoculates QZ227

| Character | QZ227 |
| --- | --- |
| Species | *Lactobacillus plantarum* |
| Collecting location | Hualong county, Qinghai, China |
| Sample sources | Wheat landrace |
| Shape | Rod |
| Gram stain | + |
| Catalase | - |
| Gas from glucose | - |
| Fermentation type | Homo |
| Growth at temp(℃): |  |
| 5 | + |
| 10 | + |
| 45 | + |
| 50 | + |
| Growth in NaCl: |  |
| 3.00% | + |
| 6.50% | + |
| Growth in tile |  |
| 0.10% | + |
| 0.30% | w |
| Growth at pH: |  |
| 2.0 | w |
| 3.0 | + |
| 3.5 | + |
| 4.0 | + |
| 4.5 | + |
| 5.0 | + |
| 5.5 | + |
| 6.0 | + |
| 8.0 | + |
| 9.0 | + |
| 10.0 | w |

+ Positive；- Negative；w Weakly positive.

**Supporting Table S2.** The chemical composition and microbial community of raw materials prior to ensiling

|  | Wheat |
| --- | --- |
| pH | 6.57±0.17 |
| Moisture (% FM) | 63.48±0.37 |
| Dry matter (%) | 36.52±0.37 |
| Crude protein (% DM) | 4.65±0.12 |
| Crude fat (% DM) | 12.51±0.75 |
| NDF (% DM) | 50.37±2.21 |
| ADF (% DM) | 34.12±1.55 |
| Crude ash (% DM) | 7.33±0.64 |
| Organic acid (mg/g DM) | |
| Lactic acid | nd |
| Acetic acid | nd |
| Propionic acid | nd |
| Butyric acid | nd |
| Viable microbiological Counts (log CFU/g FM) | |
| LAB | 2.69±0.37 |
| *E. coli* | 1.80±0.09 |
| *F. fungi* | 3.95±0.76 |
| *S. cerevisiae* | 2.22±0.59 |
| *B. subtilis* | 1.57±0.51 |
| Aerobic bacteria | nd |
| *S. marcescens* | nd |

Data are shown as mean ± standard deviation (s.d.) from three samples; nd, not detected.
